# Supplementary material for: Hollow-core conjoined-tube negative-curvature fibre with ultralow loss
Source: Nat Commun. 2018 Jul 19;9:2828. doi: 10.1038/s41467-018-05225-1 (PMC6053410; doi:10.1038/s41467-018-05225-1)
Supplement: Supplementary file 1 — Supplementary Information [file 41467_2018_5225_MOESM1_ESM.pdf]

Supplementary Information for "**Hollow-core conjoined-tube negative-curvature fibre with ultralow loss**"

Shou-fei Gao[1], Ying-ying Wang[1*], Wei Ding[2], Dong-liang Jiang[1], Shuai Gu[1], Xin Zhang[1] and Pu Wang[1]

Supplementary Note 1: Estimation of measurement uncertainty

Fibre loss measurement was done by standard cut-back technique using a fibre-based supercontinuum source (SC, YSL Photonics, SC-5), an optical spectral analyzer (OSA, Yokogawa AQ6370D, with resolution of 2 nm and sensitivity of High 3), and a butt-coupling setup. The CTF was looped with R = 16 cm. The input butt-coupling point is steadily clamped inside a splicer (FURUKAWA Fitel s178 A). The output end of the 330 m (5 m) CTF was launched into the OSA via a magnetic clamp bare fibre adaptor (OZ Optics), ensuring no stress on the fibre. To test the stability of measurement, for the two CTF lengths, we cleaved and reconnected the output fibre end to the OSA six times each. In another test, the stabilities of the SC and OSA were also examined by recording spectra every 5 minutes for half an hour (the CTF cut-back process lasts for half an hour). As shown in Supplementary Figure 1, all the repeatedly measured spectra overlap with each other very well. The transmittance of the 325 m-long CTF was derived from these experimental data.

To evaluate the measurement uncertainty, we consider the instability introduced by apparatuses and coupling points and the inaccuracy of the fibre lengths. The former factor can be analyzed by the standard deviations (SD) of the three sets of spectra. Supplementary Figure 1 shows that the relative SD's, which are the ratios of the SD to the mean, are in the level of less than 1%. The overall relative SD of the transmittance can be estimated as the square root of the sum of the three variances (square of SD), resulting in a measurement uncertainty of $10 \cdot \log_{10}(1+\mathrm{SD}) \cdot L^{-1}$. Here, the cut-back length L = 0.325 km. With regard to the inaccuracy of the CTF length (ΔL), we estimate it to be less than 0.5 m, resulting in an additional uncertainty of $\Delta L \cdot \bar{\alpha} \cdot L^{-1}$ with $\bar{\alpha}$ being the mean of the fibre loss. Supplementary Figure 2 shows the derived fibre loss spectrum with the error estimation.

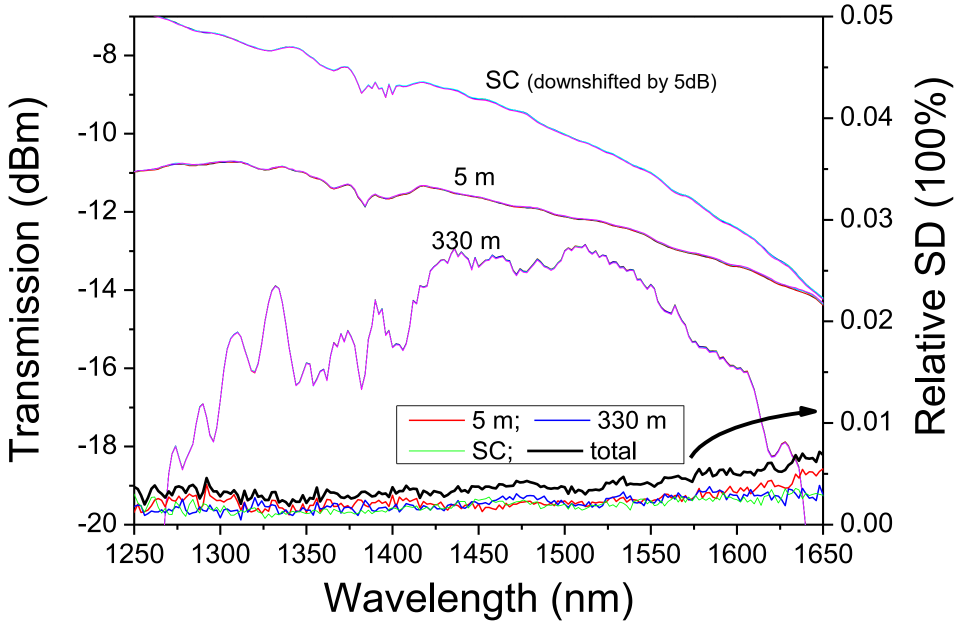

Supplementary Figure 1. Transmission spectra of the SC source, the 330 m CTF and the 5 m CTF. Here, for clarity, the spectra of the SC source have been downshifted for 5 dB. In each circumstance, 6 individual traces are acquired. The relative standard deviations (the red, blue and green curves labeled by the right axis) are analyzed from these three sets of spectra. The overall relative SD of the transmittance is shown in black color.

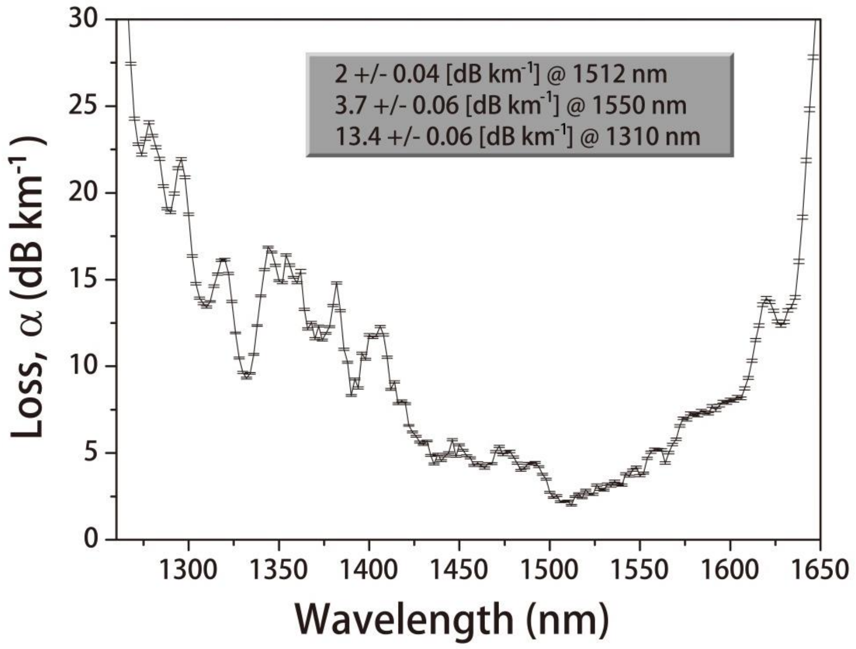

Supplementary Figure 2. Measured fiber loss (α) spectrum with error estimation. The error bars represent the measurement uncertainty, which consists of the variations of the

three sets of transmission spectra in Supplementary Figure 1 and the inaccuracy of the measured fibre length.

Supplementary Note 2: Simulation of group-velocity dispersion

Numerical simulation based on finite-element method is carried out by use of the geometrical parameters read out from the SEM image, see Fig. 1a in the main text. The resulting group-velocity dispersion (GVD, $D = -(\lambda/c)\cdot(d^2 n_{\text{eff}} / d\lambda^2)$ with n_{eff} the effective modal index [1]) curve exhibits a characteristic *S*-shaped form and a blue-shift of its zero-crossing relative to the central frequency of the transmission band due to the waveguide dispersion contribution [2], see Supplementary Figure 3. The advantageous characteristic of CTF (one type of broadband HCF) of very low chromatic dispersion is manifested with $|D| < 6$ ps nm^{-1} km^{-1} across the entire E, S, C, L telecom bands (1302-1637 nm). A number of oscillations visible in the dispersion curve can be ascribed to the undesired but minor couplings with the resonances in the cladding area. The consistence of the positions of these Fano-resonance features and the peaks in the loss spectrum, e.g. at 1278 nm, represents the fundamental Kramers–Kronig relation [3].

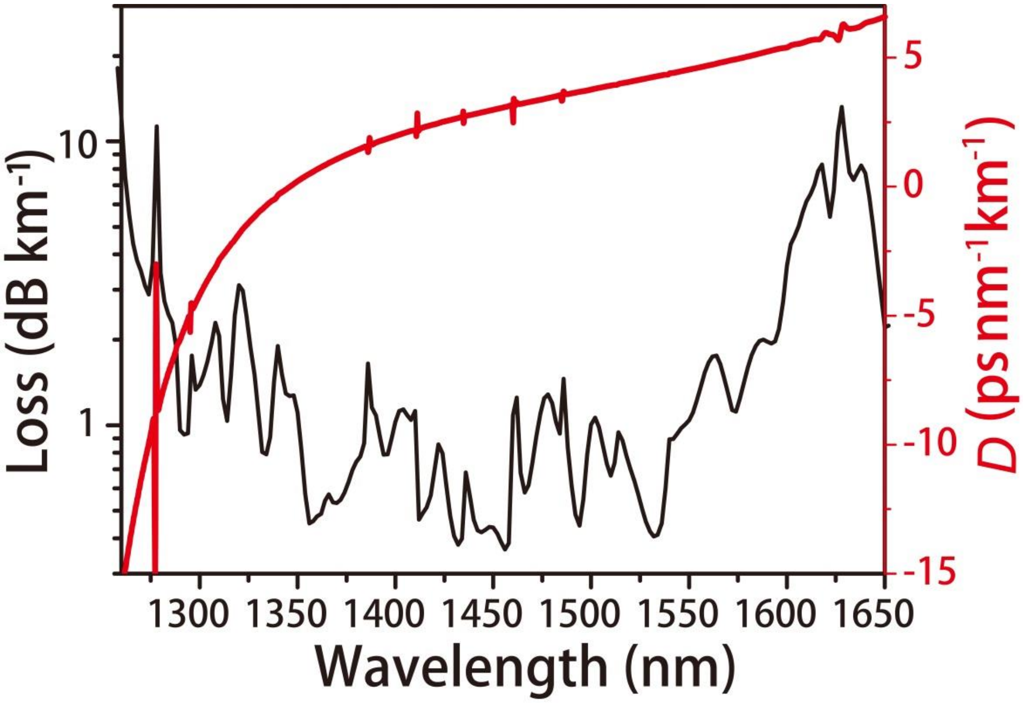

Supplementary Figure 3. Simulated loss (black solid curve) and GVD (red) versus wavelength.

Supplementary Note 3: Equivalence between D-shaped tube and circular tube

In order to directly compare with the single ring NCF structure, where the ratio of *d/D (d* being the inner diameter of the cladding tube and *D* being the inner inscribed diameter of the hollow core) is an important parameter to determine single modedness [4], we attempt to find out the equivalence between our D-shaped tube and certain circular tube with the diameter of *d'* from their dispersion curves.

The dispersion curves of the fundamental modes of the air core with the inscribed diameter of 30.5 μm and the cladding D-shaped air holes with the effective areas S_2 =335 μm^2 and S_1 = 270 μm^2 in the CTF are plotted in Supplementary Figure 4 (symbol points) using finite-element mode solver (Comsol Multiphysics). To attain the same Re(n_{eff}), analytically calculated dispersion curves of the airy modes of circular tubes with the diameters of *D'* = 32.75 μm, d'_1 = 17.5 μm and d'_2 = 20 μm and the glass thickness t = 1.12 μm are also plotted as lines in Supplementary Figure 4, overlapping very well with the former. In consistency with the definition in [4], the *d'/D* ratios in this CTF are 0.57 and 0.66.

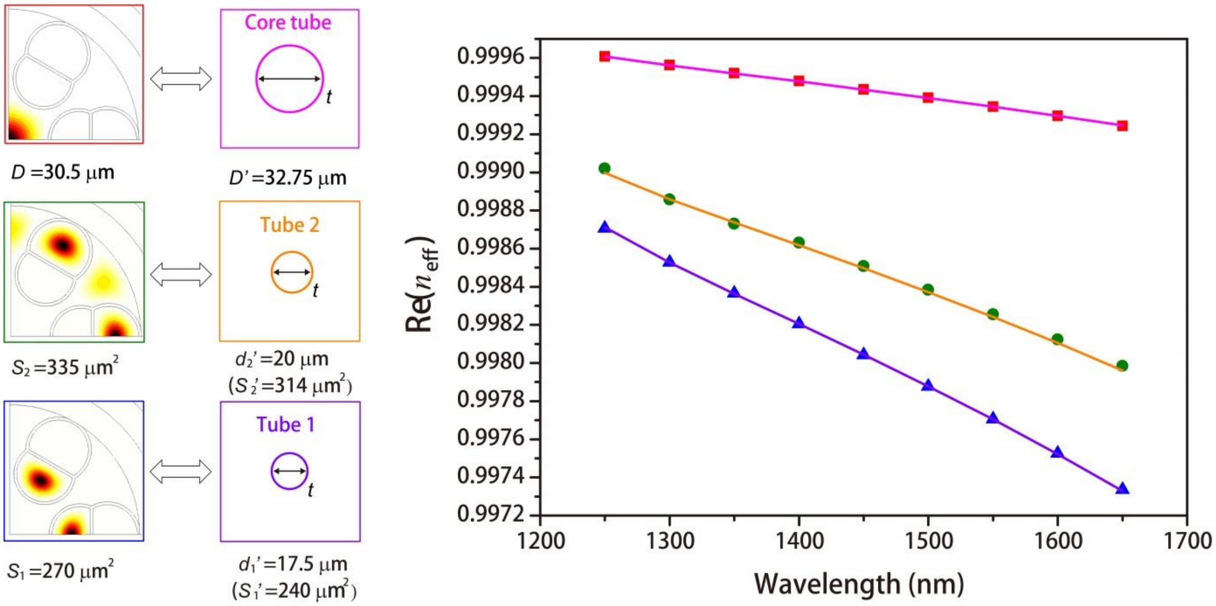

Supplementary Figure 4. Simulated dispersion curves for the fundamental modes in the CTF core (red square points), CTF cladding air hole S_2 (green circle points), CTF cladding air hole S_1 (blue triangle points) and analytically calculated dispersion curves for the airy

fundamental modes in equivalent core tube (pink line), tube 2 (orange line) and tube 1 (purple line).

Supplementary Note 4: Analytical estimation of confinement loss

Utilizing a recently developed multi-layered model [5,6], we can quantitatively estimate the confinement loss of an annular or Bragg HCF. In the circumstance of the core diameter ($2a$) being 20 times the wavelength (λ), the glancing angle of the meridional rays inside the fibre can be approximately expressed as $\theta_z = \sin^{-1}(\sqrt{1-n_{eff}{}^2}) \approx \sin^{-1}(u_{01}\lambda/2\pi a)$ for the fundamental core mode, where u_{01} = 2.405 is the first null of the zeroth-order Bessel function. Adding one glass membrane introduces two more glass:air interfaces and two more dielectric layers (one glass layer and one air layer). The confinement loss will decrease by $\left(\frac{\sqrt{n^2-1}}{4\sin\theta_z}\right)^2 \cdot \left(\frac{1+n^{2N-4}}{1+n^{2N}}\right) + 12\ \mathrm{dB}$.

Here, $n \approx 1.45$ is the refractive index of silica, the number of glass:air interfaces, N, is set to be 6, and the 12 dB represents the contribution from the anti-resonant reflecting optical waveguide (ARROW) effect [7,8] of the two added dielectric layers [3]. With $2a = 20\lambda$, above formula quickly gives a 22.4 dB of confinement loss reduction.

Above multi-layered model provides a simple but insightful picture for NCF and CTF. Inserting one glass bar into each cladding tube, which converts the ordinary tubular NCF to our 1-bar CTF, is equivalent to adding one glass membrane in fiber's radial direction. After optimizing parameters of the conjoined tubes, we obtain a simulated confinement loss reduction of 1-bar CTF of ~ 20 dB as shown in Fig. 4 (see main text), agreeing very well with the above prediction of 22.4 dB. This result corroborates that in the CTF structure Fano-resonance induced loss plays a minor role, which is a prerequisite of ultralow-loss ARF.

Supplementary References:

[1] G. P. Agrawal, Nonlinear Fiber Optics, 5th ed. (Academic Press, 2013).

[2] F. Benabid and P. J. Roberts, Linear and nonlinear optical properties of hollow core photonic crystal fiber, *J. Mod. Opt.* **58**, 87–124 (2011).

[3] W. Ding and Y. Y. Wang, Hybrid transmission bands and large birefringence in hollow-core anti-resonant fibers, *Opt. Express* **23**, 21165-21174 (2015).

[4] P. Uebel, M. C. Günendi, M. H. Frosz, G. Ahmed, N. N. Edavalath, J. Ménard and P. St. J. Russell, Broadband robustly single-mode hollow-core PCF by resonant filtering of higher-order modes. Opt. Lett. 41, 1961-1964 (2016)

[5] Y. Y. Wang and W. Ding, Confinement loss in hollow-core negative curvature fiber: A multi-layered model, *Opt. Express* **25**, 33122-33133 (2017).

[6] D. Bird, Attenuation of model hollow-core, anti-resonant fibres, *Opt. Express* **25**, 23215–23237 (2017).

[7] J.-L. Archambault, R. J. Black, S. Lacroix, & J. Bures, Loss calculations for antiresonant waveguides, J. Light. Technol. 11, 416–423 (1993).

[8] N. M. Litchinitser, A. K. Abeeluck, C. Headley, and B. J. Eggleton, Antiresonant reflecting photonic crystal optical waveguides, *Opt. Lett.* **27**, 1592–1594 (2002).
